# Breakdown in seasonal dynamics of subtropical ant communities with land-cover change

**DOI:** 10.1098/rspb.2023.1185

**Published:** 2023-10-11

**Authors:** Jamie M. Kass, Masashi Yoshimura, Masako Ogasawara, Mayuko Suwabe, Francisco Hita Garcia, Georg Fischer, Kenneth L. Dudley, Ian Donohue, Evan P. Economo

**Affiliations:** ^1^ Biodiversity and Biocomplexity Unit, Okinawa Institute of Science and Technology Graduate University, Onna, Okinawa, Japan; ^2^ Environmental Science and Informatics Section, Okinawa Institute of Science and Technology Graduate University, Onna, Okinawa, Japan; ^3^ Macroecology Laboratory, Graduate School of Life Sciences, Tohoku University, Sendai, Miyagi, Japan; ^4^ Zoology, School of Natural Sciences, Trinity College Dublin, Dublin, Republic of Ireland

**Keywords:** insect, monitoring, Japan, subtropics, temporal, variability

## Abstract

Concerns about widespread human-induced declines in insect populations are mounting, yet little is known about how land-use change modifies both the trends and variability of insect communities, particularly in understudied regions. Here, we examine how the seasonal activity patterns of ants—key drivers of terrestrial ecosystem functioning—vary with anthropogenic land-cover change on a subtropical island landscape, and whether differences in temperature or species composition can explain observed patterns. Using trap captures sampled biweekly over 2 years from a biodiversity monitoring network covering Okinawa Island, Japan, we processed 1.2 million individuals and reconstructed activity patterns within and across habitat types. Forest communities exhibited greater temporal variability of activity than those in more developed areas. Using time-series decomposition to deconstruct this pattern, we found that sites with greater human development exhibited ant communities with diminished seasonality, reduced synchrony and higher stochasticity compared with sites with greater forest cover. Our results cannot be explained by variation in regional or site temperature patterns, or by differences in species richness or composition among sites. Our study raises the possibility that disruptions to natural seasonal patterns of functionally key insect communities may comprise an important and underappreciated consequence of global environmental change that must be better understood across Earth's biomes.

## 1. Introduction

Insects comprise 95% of described terrestrial animal species on Earth and are key drivers of a multitude of ecosystem functions and services, including pollination, food provisioning, pest control, water filtration, carbon sequestration and decomposition [1]. Mounting concern about global insect declines due to human-induced environmental change presents a serious threat to a sustainable future for humanity [2–4]. However, less is known about how anthropogenic stressors change intra-annual patterns of variability and seasonality, which are also crucial for ecosystem functioning and services. Such dynamics can only be detected by sampling at fine temporal resolutions [5]. For example, the majority of studies on community temporal variability compare measurements among years and rarely within them (e.g. [6–9]), meaning that modification of seasonality due to anthropogenic factors may be overlooked. In addition to declines in insect populations, loss of community seasonal patterns via human pressures can degrade ecosystem functions and services [10,11]. For example, Hung *et al*. [12] found that habitat fragmentation via urbanization reduced seasonal turnover in bee assemblages linked to pollination provision. Therefore, better understanding is needed of how fine-scale temporal patterns of insect communities are affected in this era of accelerating global change.

Although moderate land-cover heterogeneity can benefit some insect groups, intensive land-cover modification typically has negative effects on insect diversity (e.g. [13–15]). Corresponding effects on temporal community dynamics such as seasonality are, however, less well documented. There is evidence that human development can erode seasonality of aquatic insects [16], bees [12], bugs and leafhoppers [17], and butterflies [18]. For most of these studies, loss of seasonality is linked to biotic homogenization, whereby communities become more similar and generalist due to replacement of native species with exotics [19]. As maintenance of natural seasonal patterns is associated with more reliable provision of ecosystem functions and services [11], it is important to know whether insect communities affected by human development and resulting invasions of alien species retain these seasonal patterns. This question remains unanswered, especially for understudied tropical and subtropical regions that are undergoing particularly rapid land-cover change [20], and also for islands, which have restricted land area, frequent introductions of alien species and exposure to increasingly extreme weather events [21].

Here, we examine the effects of anthropogenic land-cover change on the fine-scale temporal variability of ant community activity on subtropical Okinawa Island in southern Japan. As ants are ecologically abundant and important scavengers, predators, decomposers and mutualist partners, the overall levels of ant activity should be highly consequential for ecosystem functioning [22,23]. Moreover, given their ubiquity and high abundance [24], ants are also commonly used as bioindicators for ecosystem change [25]. Ant activity often varies throughout the year, responding to both abiotic (e.g. temperature and precipitation) and biotic (e.g. plant phenology, resource availability) factors (e.g. [26–29]). Here, ‘activity' is measured by the number of individuals intercepted in passive traps in a given time period. The realized activity patterns reflect both species abundances and differences in ‘per individual' behaviour that may vary throughout the year (this is sometimes called ‘activity density'; [30]). For example, ants may choose to forage in favourable times throughout the year, and otherwise reduce activity when conditions are unfavourable [28,31]. Moreover, the numbers of both individual worker ants and colonies can vary throughout the year [32,33]. On longer, interannual timescales, changes in activity patterns should mostly reflect population dynamics, including stochastic fluctuations or directional trends. The seasonality of activity patterns, and thus species detected in surveys, are also known to vary by vertical stratum [34], reflecting microvariation in both abiotic and biotic factors [35]. These activity patterns also modulate the interactions of ants with other organisms, such as the timing of seed dispersal and pollination [36,37].

However, very little is understood about how land-cover change affects ant community activity dynamics and seasonality in general. The seasonality question becomes especially relevant in biomes outside the temperate zone with less pronounced temperature oscillation. If temperature does not go below minimum physiological limits for foraging activity, in principle, activity could vary from nearly aseasonal to highly seasonal. That said, even in the absence of temperature seasonality, ants and other organisms can exhibit significant seasonality in tropical and subtropical regions due to some other seasonal forcing [26,38], and can have otherwise complex dynamics unrelated to seasonal climate oscillation [39].

There are reasons *a priori* to expect that forest degradation could either increase or decrease community variability. Variability can be expressed in different components; in addition to seasonal cycles, there can be stochastic fluctuations and directional trends. For ants on islands, forest degradation is associated with a higher number of alien species [40–42], including our focal area of Okinawa Island [43–45]. Such communities of recently co-occurring species may be highly unstable, and invasive ants are well known to undergo boom and bust cycles [46]. At the same time, it is possible that disturbed habitats harbour ecologically simplified communities with a common set of species that are more stable over time. Such differences may be found in the stochastic and directional components of variability (i.e. trend). In terms of seasonality, there could be thermal differences between forested and degraded habitats, causing open habitats to be warmer [47]. However, differences in the availability of sugar or protein food sources [48,49] or yet other unanticipated factors must also be considered. Of course, another possibility is that none of these factors are relevant and there are few differences in variability across habitats.

To assess how ant community dynamics vary across a gradient of habitat degradation, we used high-resolution (2-week interval) monitoring data on species- and community-level ant activity taken over 2 years on Okinawa Island. We examine whether, and how, the temporal variability of ant communities varies with anthropogenic land-cover change on a heavily populated subtropical island. We then determine the temporal components of variability (i.e. seasonality, trend or stochasticity) that contribute the most to any differences across the land-cover gradient (electronic supplementary material, figure S1). As differences in regional and *in situ* temperatures or community composition among sites can also act as possible drivers of community variability, we additionally examined their influence on variability differences. Furthermore, we assessed whether differences in temporal dynamics between habitats was due to differences among species occurring in different habitats, or due to changes within the same species occurring across habitats. Finally, we examined the responses of native and non-native species separately to determine their respective contributions to community dynamics.

## 2. Material and methods

### (a) Study sites and land-cover data

**Figure 1.**
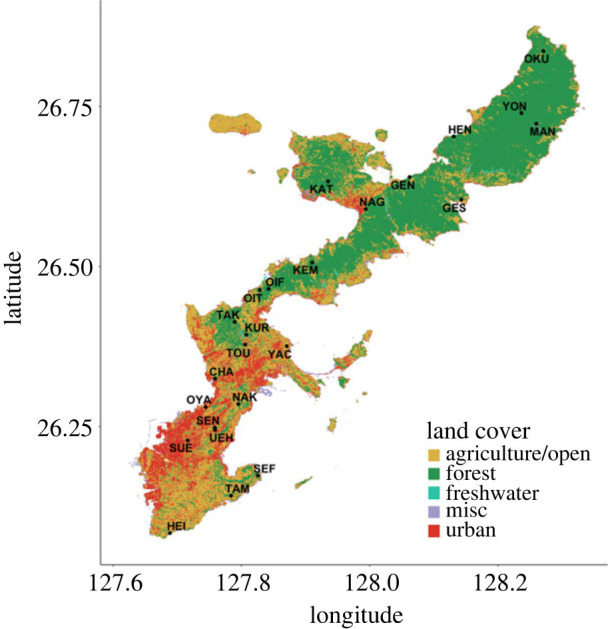
Major land-cover classes of Okinawa Island with locations of the OKEON monitoring stations, which cover the full gradient of human development across the island. Ants were sampled from three stations at each sampling site biweekly from 2016 to 2018 (total of 52 sampling periods).

Our study focuses on the subtropical main island of Okinawa in the southern Ryukyu archipelago of Japan. The island has a historical land-cover gradient, spanning from the minimally developed north to the more urban south (figure 1). Okinawa's extensive Yambaru Forest in the north was recently recognized as part of Japan's newest Natural World Heritage Site for its high endemism and biodiversity. Although the island is only 0.3% of Japan's total land area, it provides habitat to more than one-third of the country's ant species [50,51]. The few studies of ant communities in Okinawa include biodiversity surveys (e.g. [52]) and comparisons between native and alien ant populations in Yambaru [43,45,53]. This study, the first to sample island-wide ant diversity and temporal patterns in Okinawa, uses data from a biodiversity monitoring system covering the land-cover gradient of the island, administered by the Okinawa Environmental Observation Network (OKEON) Churamori Project (https://okeon.unit.oist.jp/).

We used a land-cover map for Okinawa (year 2015) to calculate representative land-cover values for the 24 OKEON monitoring sites. The sites (pairwise distances: minimum = 389 m, mean = 33 km, maximum = 102 km, standard deviation = 23 km) are located in or near forested areas across multiple land-cover types varying from broadly contiguous forest to highly agricultural or urban areas (figure 1). To characterize each site, we calculated the proportion of seven main land-cover classes (forest, agriculture, urban, grass, sand, freshwater, miscellaneous) around circular buffers with radius 1 km [54]. We chose buffers of this size to characterize representative land-cover values for areas surrounding sites (e.g. to differentiate a forest patch in an urban landscape from a large, contiguous forest), yet avoid including distant regions not representative of the sampled site. Sites near the coast included ocean area within the buffer, so we used relative proportions for all sites to consider only land area. As correlations were high between the main land-cover classes of interest (that is, forest, agriculture and urban; electronic supplementary material, table S1), we used principal component analysis (R function *prcomp*) after applying an arcsine transformation to the proportions to improve normality (e.g. [55]) and retained the first and second axes (variance explained: PC1 = 81% and PC2 = 11%) for use as explanatory variables. PC1 represents the forested (high) to developed (low; urban and/or agriculture) gradient, while PC2 represents the rural (high; agriculture and/or grass) to urban (low) gradient (electronic supplementary material, figure S2). All analyses were conducted in R [56], and spatial analyses were conducted with R packages *sf* [57] for vector data and *raster* [58] for gridded data.

### (b) Ant activity data

Insect count data sampled with passive traps are usually interpreted as a measure of ‘activity', or the rate at which individuals intersect a point in space, rather than direct estimates of abundance. For example, if ant count is higher in one area than another, this is likely a combination of the time spent foraging, rate of movement and overall abundance—this quantity is referred to as ‘activity’. Because of this definition, differences in abundance are generally reflected in ant activity data [59].

We sampled worker ant activity biweekly with Sea, Land and Air Malaise (SLAM) traps for 2 years and identified samples to the species level. The ant fauna of Japan is particularly well documented, thus allowing us to use species identification data of the highest quality. Ant specimens were identified by several ant taxonomists based on qualitative morphology by using regional identification keys (e.g. Japanese Ant Image Database: http://ant.miyakyo-u.ac.jp/E/index.html), comparisons with local reference collections or online image databases (Antweb: https://www.antweb.org). Although Malaise-type traps are often used to catch flying insects, they can also be used to catch a variety of non-flying insects [60], including a wide diversity of worker ants [41,61]. We chose to use SLAM traps as ground pitfall traps tend not to work in moist forest environments like Okinawa, as they quickly fill with snails, and Winkler extractions are too destructive to employ on a biweekly repeated basis. Like any entomological survey method, SLAM traps provide a biased sample of the community, in this case towards ants walking on low vegetation. However, this is a consistent bias and no different from any trapping or detection method in ecology that targets a stratum of an ecosystem (e.g. leaf litter, canopy, soil, etc.). Our goal here is to examine differences in community dynamics across habitats, not estimate absolute abundances of species in the ecosystem. Each OKEON site has three stations (pairwise distances between stations at a site: mean = 90 m, minimum = 19 m, maximum = 195 m, standard deviation = 45 m) with one SLAM trap (Large BT1005, MegaView Science Co., Ltd.) each; we sampled each trap every 2 weeks from March 2016 to March 2018 (further details in electronic supplementary material, A). The trap placement represented the local-scale habitat within the site area. In most cases, this results in either forest or open cover for all three traps, but for more transitional/mixed habitats, there may be variation in shading among traps.

We additionally categorized all species by alien status as either native, alien, or uncertain based on expert opinion and biogeographic databases [50,62,63] (https://www.antwiki.org/, https://www.antweb.org/). Native refers to species that are based in the Ryukyu region as understood by current taxonomic and biogeographic knowledge, and that have no evidence of human transfer. Alien refers to species that definitively have native ranges in different parts of the world (e.g. South America, Africa) and have dispersed to Okinawa via human activity. Uncertain species lack sufficient data to make such determinations, usually because they are species native to the Asian region but known to be transported by human commerce. In such cases, it is difficult to say for certain whether they are actually native to Okinawa or transported there by humans (or both).

**Figure 2.**
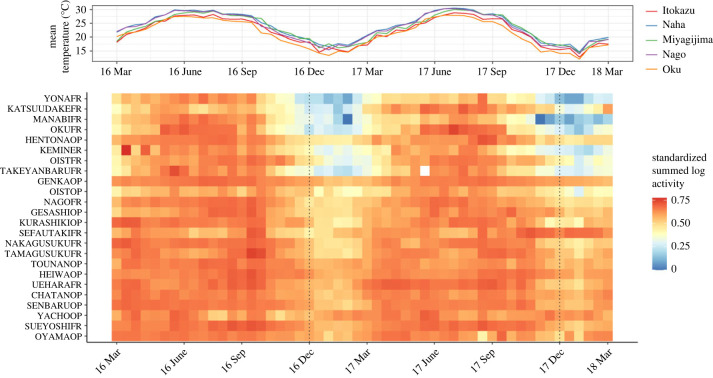
Heat map showing the standardized ant activity (i.e. logs of summed site counts divided by the log of the grand total count) over 2 years in Okinawa (bottom). Mean temperature measurements (°C) from the Japan Meteorological Agency across the island, ordered from south to north, are included for reference (top). Sites are ordered by values of land-cover PC1, explaining the anthropogenic stress gradient (top: more forested, bottom: more developed (urban and/or agriculture)). Dotted lines show the last sampling period before the new year.

Some prevalent species had extreme count outliers, such as *Technomyrmex brunneus* (representing, for example, 2-week counts greater than 5000), likely caused by unusual events such as moving to nests nearby or intense foraging at specific food sources rather than overall trends in activity patterns. As these rare events could have a large effect on variability estimates, we explored thresholding by truncating large values to a maximum number. Ultimately, we limited count values to a threshold of 500 individuals for a given species at a station within a 2-week period—such records with a count of over 500 correspond to the most extreme 1% of values. While arbitrary, we explored a range of threshold values and did not find sensitivity of our results to threshold value. For each species during each sampling period, we summed counts over the three stations per site to determine site-level activity (figure 2), and over all sites per land-cover group for grouped species-level activity (see section d). We then calculated total site activity as the sum of all counts per site.

Sampled abundance (or activity) and variability can be correlated due to sampling error alone because of mean-variance scaling [64]. For example, even if species dynamics are identical across two sites, if we sample fewer individuals from site A than site B (e.g. due to effects of sampling station placement), site B may have a higher temporal variability simply because its counts are higher. While in some sense this could reflect the actual variability of species encounters at specific locations (that is, at the stations), we wanted to disentangle differences in community variability from those attributable to sampling error. Thus, we rarefied the data from all sites so that the total count (sum of all samples across the two years) for each site equals the total count at the site with the least activity (electronic supplementary material, figure S1, B; [65]). Finally, to determine whether species richness differences could drive any observed differences in temporal variability, we calculated site-specific total species richness, and the richness of each alien status group from the observed and rarefied data, making extrapolations to account for incomplete sampling using Hill numbers (*q* = 0) with the R package *iNEXT* 3.0.0 [66].

### (c) Temporal variability and time-series decomposition

We calculated two aspects of temporal variability: functional and compositional. Functional variability refers to activity changes at the aggregate scale that approximate the variability of ecosystem processes and functions produced by a community [67], and we calculated this as the coefficient of variation (ratio of the standard deviation to the mean) of summed ant counts across all species per site [64]. Compositional variability estimates the variation in community composition over time, or temporal beta-diversity, and we calculated this as the total variability of the species composition matrix (species × time step) after a Hellinger transformation (to ensure purely relative count data; [68]) using the function *beta.div* from the R package *adespatial* [69]. As an additional product of this function, we derived species contributions to beta diversity per site, calculated as species variance divided by the total community variance (summing to 1). We then summed these values for each alien status category to determine their contributions. As we use ant count data, compositional changes would thus represent changes in relative activity rates of different species in the local species pool, which in turn is a function of the abundance and patterns of behaviour [59]. We calculated both indices of temporal variability on each rarefied site activity dataset, then found the mean values across datasets. These mean rarefied site values were used in subsequent models.

Next, we used temporal decomposition models to determine the relationships of temporal variability components with observed patterns. Time series can be decomposed into components describing different underlying temporal patterns in the data: ‘seasonal' processes that correspond to periodic patterns and events that occur on a yearly cycle, ‘trend' that describes directional change, and ‘remainder' that represents any residual variation not captured by the other components (i.e. short-term stochastic fluctuations; [70]). A community with high temporal variability may have strong cyclical patterns, increasing or decreasing trends, high stochasticity or some combination of these. For temporal datasets spanning many annual cycles, models that can estimate complex seasonal responses (e.g. wavelet analysis; [5]) are often employed, but for studies spanning few annual cycles at a fine temporal resolution, simpler models with fewer assumptions and problems with overfitting are preferable. We estimated additive temporal components for the ant activity data by fitting time-series linear models with temporal predictors using the R package *fable* [71]. As our time series was short, we used simple predictors consisting of a linear trend and a seasonal signal approximated with an annual Fourier term with the simplest maximum order (*K*) of 1—this models seasonality as a sine wave [70]. We fitted this model to the rarefied data at both the site-level and species-level for land-cover groups (see Section d), then decomposed the count value at each time-step. For each site, we measured the absolute variance of each temporal component, but also the relative component variance, defined here as the individual component variance divided by summed variance of all components (electronic supplementary material, C).

We used simple linear models to estimate relationships between land cover and our focal metrics of temporal variability. Specifically, we fitted models for the natural log of total ant activity (before rarefaction), functional and compositional temporal variability, and absolute and relative variances of time-series components. To explore differences between native and alien species, we also fitted models separately by alien status for site richness and summed species' contributions to beta diversity. As some OKEON sites are relatively close to others, we assessed spatial autocorrelation in our model residuals by calculating Moran's I using the site coordinates (Universal Transverse Mercator (UTM) projection) and testing with random permutations using the function *moran.randtest*() from *adespatial* (999 repetitions; [69]). As this test was not significant for any of the models (*p* > 0.05), we did not use more complex generalized least-squares (GLS) models with spatial structure. We performed model selection on linear combinations of the predictor variables land-cover PC1 and PC2 using the Akaike Information Criterion corrected for small sample sizes (AICc).

### (d) Assessing effects of site differences in temperature and community composition on observed seasonality patterns

To determine if other differences among sites may be responsible for any observed variation in ant community activity and relationships with land cover, we additionally examined differences in (1) regional and site-level temperature and (2) tested whether seasonality differences persisted after standardizing community composition between land-cover groups. If annual temperature patterns differ considerably from north to south on the island, or across sites due to differential trap placement, this can affect observed ant activity patterns. At the same time, if particular communities at certain sites have uniquely low or high seasonal patterns, for example, compositional differences across sites can also affect observed patterns. For (1), we downloaded regional climate data for the collection period from the Japan Meteorological Agency (JMA) database (http://www.jma.go.jp/en/amedas_h/map65.html, accessed 4 January 2020) and calculated temperature means and extremes from the six climate stations on Okinawa Island that collect temperature data (Miyagijima, Itokazu, Naha, Ashimine, Nago, Oku). We also collected *in situ* site-level data throughout the collection period at one station per site to characterize local air and soil temperature patterns (WatchDog 2900ET Weather Station 1.5 m above ground and SMEC WaterScout 300 Soil Sensor 10 cm below ground, Spectrum Technologies). With the same workflow as for the ant activity data, we calculated absolute variance of seasonality for regional and site-level temperature time-series, then estimated relationships with land-cover PC1 using linear models.

For (2), we conducted analyses to test whether differences in community composition alone, without variation in seasonality within species, could be driving the observed patterns in seasonality, or alternatively if habitat-dependent dynamics of species occurring in both habitat types were responsible. To do this, we compared the relative seasonality variance of species shared between two representative land-cover groups: ‘forested’ and ‘developed'. If species generally have the same seasonality patterns between land-cover groups, any differences in seasonality we observed would be due to compositional differences among sites and not differences in land cover. We partitioned the sites into these two groups of eight sites each using land-cover PC1 and retained only those species shared between groups and with total counts greater than 100. The ‘developed' group comprised sites with more agricultural and/or urban land-cover. We excluded the eight sites with intermediate values of PC1 to better represent the most characteristic sites for each land-cover group. To make balanced comparisons between land-cover groups, we rarefied each species' activity data to the minimum count of that species between groups (electronic supplementary material, figure S1, B). To determine whether community composition differences affected our results, we fitted time-series linear models to each species in the land-cover groups and calculated the relative variance of their seasonality (forested versus developed sites). As differences between the land-cover groups were not normally distributed (Shapiro–Wilk test; *p* = 0.011), we used the non-parametric Wilcoxon signed-rank test to determine whether the same species between groups exhibit significant differences in relative seasonality variance. Additionally, to determine how temporal mismatch changes with alien status, we calculated the synchrony of seasonality for each alien status between land-cover groups and standardized them to the same scale. We used the R package *codyn* 2.0.5 [72] to calculate synchrony using the method described by Loreau & De Mazancourt [73]. We then compared species' seasonal curves to the average seasonality curve for each land-cover group to visualize the degree of temporal mismatch by alien status.

## 3. Results

**Figure 3.**
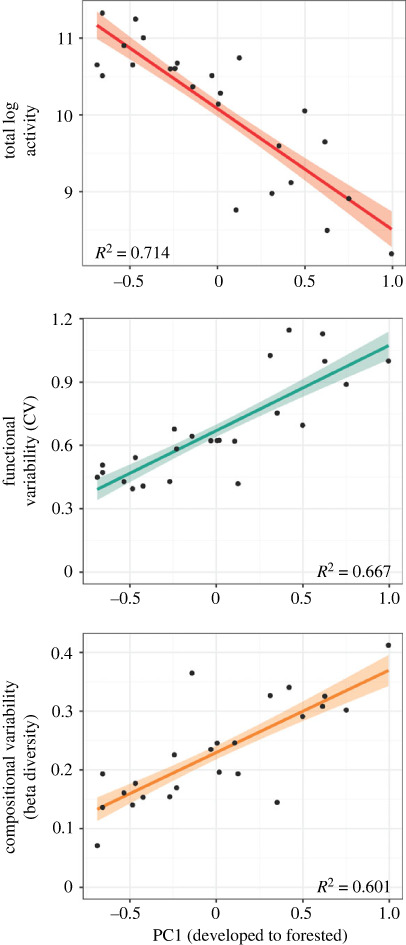
Trends in total log activity, functional variability and compositional variability for ants across a gradient of anthropogenic impact. Relationships with PC1, explaining the anthropogenic stress gradient (low: more developed (urban and/or agriculture), high: more forested). Total log ant activity (before rarefaction) correlates negatively with PC1, while indices of temporal variability (functional: coefficient of variation, compositional: beta diversity) correlate positively.

Our sampling for 2016–2018 resulted in the recovery of 1 378 324 classified individual workers (before thresholding) across 91 ant species. Total count was less than five individuals for 21 species and greater than 10 000 individuals for 15 species, with a minimum of 1 (*Aenictus ceylonicus*, *Crematogaster* cf. *matsumurai*, *Ectomomyrmex* sp*.*, *Erromyrma latinodis*, *Hypoponera* sp., *Leptogenys confucii*, *Strumigenys hirashimai*, *Strumigenys mazu*) and a maximum of 259 180 (*Tetramorium bicarinatum*). The pattern of ant activity dropping in the winter and rising in the summer qualitatively diminished as human disturbance increased (figure 2). Total ant activity (after thresholding to 500) had a nearly 23-fold difference between the site with the minimum count (Yona Forest, *n* = 3616) and that with the maximum count (Sueyoshi Forest, *n* = 82 781). The natural log of total activity had a strong negative relationship with land-cover PC1 (*R*^2^ = 0.71, *p* < 0.001), indicating that ant activity overall was higher in developed areas than forested areas (figure 3). For all subsequent analyses, rarefaction resampled all sites to the minimum site count.

**Figure 4.**
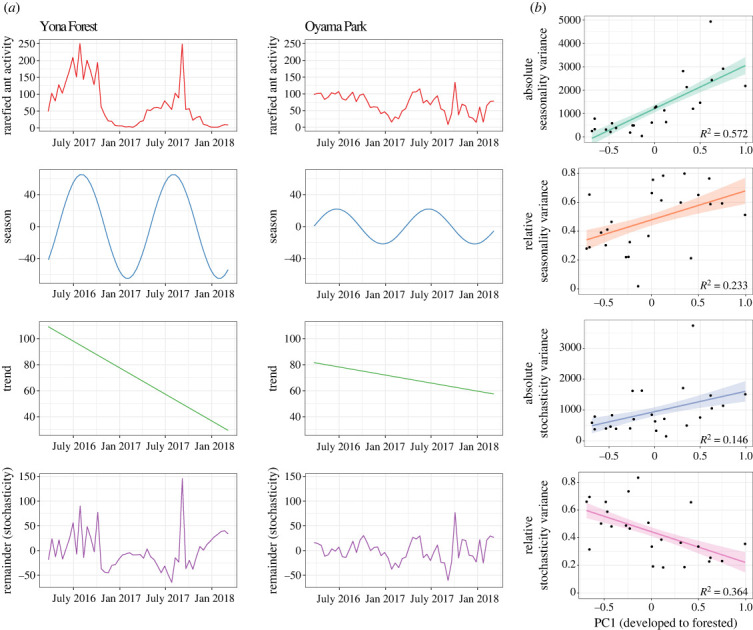
(*a*) Time-series decomposition of rarefied ant activity for the sites with the most forest (Yona Forest) and most human development (Oyama Park) within a 1 km buffer. (*b*) Relationships between absolute and relative variance of seasonality and stochastic temporal components and PC1 across all 24 sites, explaining the anthropogenic stress gradient (low: more developed (urban and/or agriculture), high: more forested). Relative variance here is calculated as individual component variance divided by summed variance of all components.

The linear models suggested strong relationships with land-cover PC1 for most metrics of temporal variability (figures 3 and 4). Based on model selection via AICc, all such models included PC1 as the sole predictor except the relative variances of seasonality and stochasticity, which also used PC2 (electronic supplementary material, table S3). Functional and compositional temporal variability both had strong positive relationships with PC1 (functional: *R*^2^ = 0.67, *p* < 0.001; compositional: *R*^2^ = 0.60, *p* < 0.001; figure 3). The species' contributions to beta diversity (electronic supplementary material, figure S3) that explained individual species' impacts on compositional variability were also positively correlated with PC1 for native species (*R*^2^ = 0.40, *p* < 0.001), but uncertain species (*R*^2^ = 0.28, *p* < 0.01) had a weaker, negative relationship (alien species showed no relationship: *R*^2^ = 0.08, *p* > 0.05). Absolute seasonality and stochasticity variance had positive relationships with PC1, though that for seasonality (*R*^2^ = 0.57, *p* < 0.001) was stronger than that for stochasticity (*R*^2^ = 0.15, *p* < 0.05; figure 4). On the other hand, relative seasonality variance (*R*^2^ = 0.23, *p* < 0.05) was positively related to PC1, while relative stochasticity variance (*R*^2^ = 0.36, *p* < 0.01) had a negative relationship (both had *p* > 0.05 for PC2; figure 4). All linear regression results are found in electronic supplementary material, table S3.

**Figure 5.**
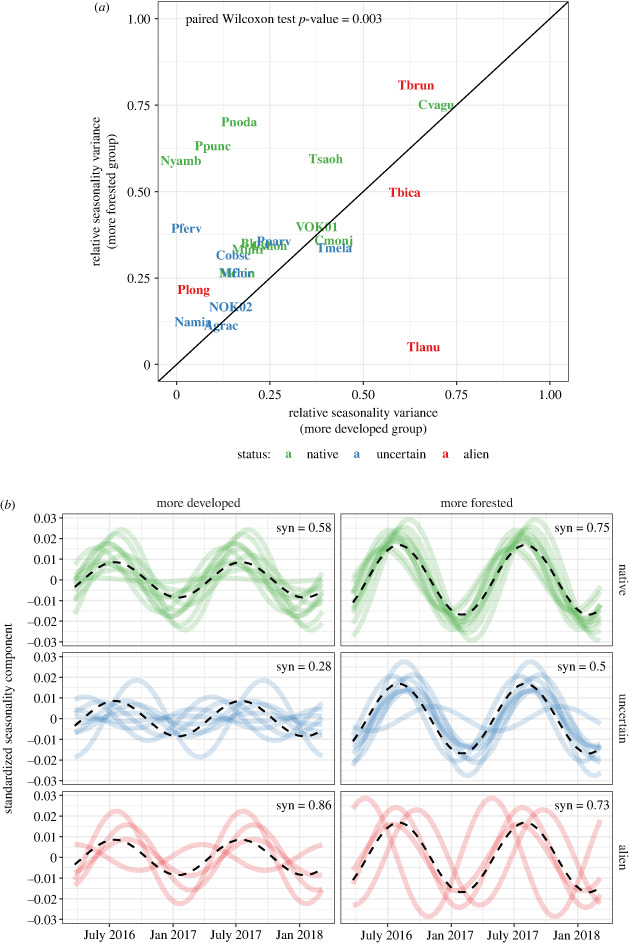
(*a*) Relative seasonality variance calculated per ant species in two representative land-cover groups each containing eight sites: more forested and more developed. Only species shared between groups (abbreviations of those found in electronic supplementary material, table S2) and with total counts greater than or equal to 100 were retained, whereupon species counts were rarefied to the minimum between groups. On average, species have a higher relative seasonality variance in the forested group (paired Wilcoxon Test; *p* = 0.003), and no apparent patterns exist regarding alien status. (*b*) Seasonal component time-series for each species (lines) found in the more forested and more developed groups, standardized by total species activity and separated by alien status. The dashed black line shows the mean standardized seasonality across all species per land-cover group, showing much greater temporal mismatch in ant communities at sites with greater human development.

We found no evidence that differences in species richness, temperatures at different scales or species compositions are candidates as drivers of observed differences in ant community temporal variability. We found no relationship between PC1 and total species richness (either observed or extrapolated), and a weak correlation with rarefied richness (*R*^2^ = 0.15, *p* < 0.05) due to the loss of rare species during the rarefaction process, though we consider this unlikely to bias our results (electronic supplementary material, figure S4). We did find strong positive relationships with PC1 for native richness values (observed: *R*^2^ = 0.47, *p* < 0.001; extrapolated: *R*^2^ = 0.35, *p* < 0.005; rarefied: *R*^2^ = 0.61, *p* < 0.001) indicating that more native species can be found in the more forested sites, and weaker negative relationships with richness for uncertain (observed: *R*^2^ = 0.32, *p* < 0.005; extrapolated: *R*^2^ = 0.33, *p* < 0.005; rarefied: *R*^2^ = 0.33, *p* < 0.005) and alien species (observed: *R*^2^ = 0.29, *p* < 0.005; extrapolated: *R*^2^ = 0.20, *p* < 0.05; rarefied: *R*^2^ = 0.20, *p* < 0.05) (electronic supplementary material, table S3, figure S4). Moreover, we found no clear patterns in the absolute variance of regional temperature seasonality from JMA climate stations spanning the island from north to south, nor did we find relationships between the absolute seasonality variance of *in situ* air or soil temperature and PC1 (electronic supplementary material, figure S5). Of the sites with at least one exposed sensor that were not located below canopy cover, two (Oyama Park and OIST Open (OYA and OIT, respectively, in electronic supplementary material, figure S5)) had relatively high absolute variances, though two had values closer to average (Kurashiki Open (KUR), Gesashi Open (GES)), and one had relatively low variance (Genka Open (GEN)). Concerning the tests of different threshold sizes, although we observed some differences, our results remained similar (electronic supplementary material, D, figure S6). Lastly, individual species showed differing seasonality between land-cover groups (more forested and more developed). Comparisons revealed that, in general, the same species have a higher relative seasonality variance at forested sites than at sites with more human development (*p* < 0.005), though the alien species *Tetramorium lanuginosum* was a notable exception (*Tlanu* in figure 5). While seasonality was stronger in the forested group for all species, when separated by alien status we observed higher synchrony in the forested group for native (*n* = 11, forested: 0.75, developed: 0.58) and uncertain species (*n* = 8, forested: 0.5, developed: 0.28), but the opposite pattern for alien species (*n* = 4, forested: 0.73, developed: 0.86) (figure 5).

## 4. Discussion

Our study sheds light on how fine-scale temporal dynamics of ant communities vary across a gradient of human-induced land-cover change in a subtropical island environment. Notably, we show that land use changes the activity dynamics of ant communities by degrading seasonal cycles. Our results for the intra-annual scale complement an extensive number of recent studies focusing on the temperate zone that examine change of insect populations over broader timescales [12,59,74–76], but also those few with enough intra-annual sampling to examine changes to seasonality [12,47]. However, those studies from the temperate zone are from ecosystems with dramatic seasonal variation in temperature that essentially forces seasonality in activity patterns. These are not representative of huge fractions of the subtropics and tropics where temperature varies meaningfully but does not enforce a shutdown of the insect community. Thus, the effect of environmental degradation on seasonality of insect populations in such environments is still not well understood [77].

Our study fills an important gap by showing that ant communities have substantially altered activity patterns across the ecological disturbance gradient. We found that temporal variability diminishes for the subtropical ant communities of Okinawa Island as land cover transitions from forested to more developed, and that this is related to a breakdown in seasonality. Forested sites were more variable overall, but their resident ant communities were more seasonal and synchronous, while community variability for developed sites (i.e. urban and agricultural) was characterized by stochastic variation. Although native ant richness was higher in forested sites, we determined that the observed differences in seasonality across the land-cover gradient were not due to differences in regional or local temperature patterns, nor to species composition among sites. Compositional differences between communities in different land-cover types, such as the prevalence of one or more highly seasonal species, could drive observed relationships between community seasonality and land cover. However, we found that populations of the same species occupying forested sites were more seasonal than those with more human development. Further, when we separated species by alien status, we found that the strong seasonal patterns we observed in the forest were more synchronous for native than alien species, though both synchrony values are reasonably high and the considerably smaller sample size for alien species should be taken into consideration.

We found a positive relationship between functional and compositional temporal variability, meaning that those sites with communities that varied more as a whole tended also to have higher total variation of the individual species in the local pool. The relationship between functional and compositional variability can range from positive to negative depending on the system and exposure to disturbance [78–80]. Two characteristics of more forested sites may help explain this positive relationship. The first is that more forested sites had higher variability in general at the biweekly timescale. The second is that, at more forested sites, native species had higher richness and the highest contributions to beta diversity. Sites with high richness that include substantial numbers of alien species can have community dynamics that differ considerably from similar sites in which native species dominate [81,82]. Thus, including alien species in richness estimates can affect how diversity–stability relationships are interpreted (e.g. [83]).

Our results raise the question of what mechanisms drive these patterns. We outline several hypotheses for why seasonal variation in activity could vary across the land-cover gradient. First, there is a possibility that ants are simply responding to the temperature they experience on a physiological level, reflecting temperature and microclimatic variation across habitats. Ants are well-known to be sensitive to temperature variation, with foraging limited to certain temperature ranges [31,84]. Regional climatic variation across the island is very modest, and with no consistent differences in seasonality. Further, we found no relationships between the seasonal variance of *in situ* air and soil temperature and the main land-cover gradient PC1. However, differences in canopy openness, vegetation and urbanization (via heat-island effects, etc.) may create conditions that are relevant for promoting and depressing ant foraging and the prevalence of such conditions might be higher in more degraded habitats. Comprehensive measurements with small sensors at various habitat strata for representative sites could help elucidate whether this phenomenon could differ across land-cover types. Another possibility is that ants are responding to resource availability patterns. In this scenario, it is possible that resources are more seasonal inside the forest rather than in more human-impacted areas. This could occur, for example, because of anthropogenic food sources that are more static throughout the year, or because of differences in phenology of vegetation inside or outside the forest [85]. A related question is whether these differences in seasonality are limited to ants or are observed across the arthropod community. Were arthropod seasonality responding more generally to land cover, this would provide a resource pattern that could affect ant activity. Related to this, it is unclear why there seems to be little seasonal niche differentiation for ant species in forested areas, which have activity patterns that follow temperature quite closely in synchrony. One possibility is that species become most active at different times during the same warm season to avoid competition—we saw some evidence of this in the standardized seasonal curves (figure 5). This could be due to the inability to achieve high activity levels during cooler months due to physiological limitations, which may be absent in more developed environments with anthropogenic temperature refugia. Other potential explanations relating to differences in the ecology or physical environments across habitat types likely exist, but more targeted research is needed to address this question, probably involving experiments to differentiate mechanisms.

Our results come with a few caveats. First, the fact that our data represent only 2 years is a limitation of our study. However, our sampling was very dense within those 2 years (24 sites, 72 traps, 52 samples per trap, > 1.2 million individuals observed), and the analytical methods we used were specifically geared toward decomposing short time-series into periodic, trend and stochastic components. Thus, we are confident that our conclusion that seasonality depends on land cover is robust for the 2 years involved. It seems unlikely that these 2 years would be outliers, with seasonality greater in forest habitats in some years but not others. However, we cannot rule this out. In any case, the broader conclusion that activity dynamics depend on land cover would still be valid. Second, seasonality of ant activity can also reflect seasonal differences in use of vertical strata, which have been shown for ants in tropical premontane [86] and dry forests [35,87]. Thus, the loss of seasonality we observed for developed areas may also represent less movement between forest strata, but testing for this would require focused experiments. Finally, it remains to be seen whether our results for ants foraging in low vegetation generalize to the entire insect community and across habitat strata.

We measured variability over 2 years, but there may be other types of variability that only manifest on longer timescales and are also correlated with land cover. For example, introduced ants are known to have boom-bust dynamics when they reach new localities [46]. Over longer timescales, communities in more human-dominated areas may prove to be more dynamic with successive shifts in the community. Indeed, we witnessed such an event in our 2-year period at one of our sites (Sefautaki Forest), where *Pheidole megacephala*, a notoriously impactful invasive ant, arrived and quickly reached high activity, raising variability of this site beyond what was typical of others with similar land-cover characteristics. Recent research in Yambaru Forest has documented resilience of native ant communities to disturbance based on surveys of invasions at developed areas after regrowth [88], so this site too may eventually recover to its previous structure. Thus, successive regime shifts in these more disturbed communities could occur over longer timescales, but only monitoring over more years would reveal such dynamics.

## 5. Conclusion

This study provides novel insight into the seasonal dynamics of subtropical ant communities across differing levels of human development. Importantly, it is the first study to our knowledge to measure seasonality of ant activity with high-frequency sampling across a human disturbance gradient. The results of our study, which link anthropogenic disturbance in the form of agricultural and urban development to a reduction in intra-annual temporal variability due to weakening seasonality, support the growing evidence that human development reduces natural seasonal patterns [12,18,89,90]. This study focuses on correlative relationships and does not identify causal mechanisms; thus, future targeted work is needed to unpack the reasons for change in temporal activity. However, it does provide a compelling demonstration that continuing habitat loss and fragmentation can lead to increasing loss of natural temporal patterns for insect communities in the parts of the globe with the highest insect diversity, the subtropics and tropics. As loss of seasonal patterns has been linked to a subsequent reduction in key ecosystem functions and services [10,11], increasing development could result in disruptions to nutrient cycling and food security [91]. To better understand the generality of these patterns, future studies using high-frequency sampling should focus on monitoring a greater diversity of insect communities across different habitat strata (i.e. arboreal, subterranean; [92]) in a variety of biomes.

The possibility of widespread insect declines is receiving a great deal of attention, with good reason. We argue that more attention on the impacts of land-cover change on insect community seasonality, and ecological seasonality more generally, should aid ongoing management and conservation efforts to help preserve the important ecosystem functions and services insects provide.

## Data Availability

All R code and data used in the analysis, including ant activity, land cover, regional island-wide climate and *in situ* site-specific climate, can be found on Zenodo and Data Dryad (all links at: https://doi.org/10.5061/dryad.zkh1893fk [93]). Supplementary material is available online [94].

## References

[1] Schowalter TD. 2013 Insects and sustainability of ecosystem services. Boca Raton, FL: CRC Press.

[2] Goulson D. 2019 The insect apocalypse, and why it matters. Curr. Biol. **29**, R967-R971. (10.1016/j.cub.2019.06.069) 31593678

[3] Wagner DL. 2020 Insect declines in the Anthropocene. Annu. Rev. Entomol. **65**, 457-480. (10.1146/annurev-ento-011019-025151) 31610138

[4] Zhou Y, Zhang H, Liu D, Khashaveh A, Li Q, Wyckhuys KA, Wu K. 2023 Long-term insect censuses capture progressive loss of ecosystem functioning in East Asia. Sci. Adv. **9**, eade9341. (10.1126/sciadv.ade9341) 36735783 PMC9897670

[5] Tonkin JD, Bogan MT, Bonada N, Rios-Touma B, Lytle DA. 2017 Seasonality and predictability shape temporal species diversity. Ecology **98**, 1201-1216. (10.1002/ecy.1761) 28144975

[6] Cottingham KL, Brown BL, Lennon JT. 2001 Biodiversity may regulate the temporal variability of ecological systems. Ecol. Lett. **4**, 72-85. (10.1046/j.1461-0248.2001.00189.x)

[7] de Mazancourt C et al. 2013 Predicting ecosystem stability from community composition and biodiversity. Ecol. Lett. **16**, 617-625. (10.1111/ele.12088) 23438189

[8] Olivier T, Thébault E, Elias M, Fontaine B, Fontaine C. 2020 Urbanization and agricultural intensification destabilize animal communities differently than diversity loss. Nat. Comm. **11**, 1-9. (10.1038/s41467-020-16240-6) PMC7264125 32483158

[9] Tilman D, Reich PB, Knops JMH. 2006 Biodiversity and ecosystem stability in a decade-long grassland experiment. Nature **441**, 629-632. (10.1038/nature04742) 16738658

[10] Ross SRP-J, Arnoldi J-F, Loreau M, White CD, Stout JC, Jackson AL, Donohue I. 2021 Universal scaling of robustness of ecosystem services to species loss. Nat. Comm. **12**, 1-7. (10.1038/s41467-020-20314-w) PMC8397752 34453056

[11] Stevenson TJ et al. 2015 Disrupted seasonal biology impacts health, food security and ecosystems. Proc. R. Soc. B **282**, 20151453. (10.1098/rspb.2015.1453) PMC4633868 26468242

[12] Hung K-LJ, Ascher JS, Holway DA. 2017 Urbanization-induced habitat fragmentation erodes multiple components of temporal diversity in a Southern California native bee assemblage. PLoS ONE **12**, e0184136. 28854229 10.1371/journal.pone.0184136 PMC5576854

[13] Corro EJ, Ahuatzin DA, Jaimes AA, Favila ME, Ribeiro MC, López-Acosta JC, Dáttilo W. 2019 Forest cover and landscape heterogeneity shape ant–plant co-occurrence networks in human-dominated tropical rainforests. Landsc. Ecol. **34**, 93-104. (10.1007/s10980-018-0747-4)

[14] Oliver I, Dorrough J, Doherty H, Andrew NR. 2016 Additive and synergistic effects of land cover, land use and climate on insect biodiversity. Landsc. Ecol. **31**, 2415-2431. (10.1007/s10980-016-0411-9)

[15] Senapathi D et al. 2015 The impact of over 80 years of land cover changes on bee and wasp pollinator communities in England. Proc. R. Soc. B **282**, 20150294. (10.1098/rspb.2015.0294) PMC4426632 25833861

[16] de Castro DMP, Dolédec S, Callisto M. 2018 Land cover disturbance homogenizes aquatic insect functional structure in neotropical savanna streams. Ecol. Indic. **84**, 573-582. (10.1016/j.ecolind.2017.09.030)

[17] Knop E. 2016 Biotic homogenization of three insect groups due to urbanization. Glob. Chang. Biol. **22**, 228-236. (10.1111/gcb.13091) 26367396

[18] Uchida K, Fujimoto H, Ushimaru A. 2018 Urbanization promotes the loss of seasonal dynamics in the semi-natural grasslands of an East Asian megacity. Basic Appl. Ecol. **29**, 1-11. (10.1016/j.baae.2018.03.009)

[19] McKinney ML. 2006 Urbanization as a major cause of biotic homogenization. Biol. Conserv. **127**, 247-260. (10.1016/j.biocon.2005.09.005)

[20] Janzen DH, Hallwachs W. 2021 To us insectometers, it is clear that insect decline in our Costa Rican tropics is real, so let's be kind to the survivors. Proc. Natl Acad. Sci. USA **118**, e2002546117. (10.1073/pnas.2002546117) 33431562 PMC7812782

[21] Russell JC, Kueffer C. 2019 Island biodiversity in the Anthropocene. Annu. Rev. Environ. Resour. **44**, 31-60. (10.1146/annurev-environ-101718-033245)

[22] Hölldobler B, Wilson E. 1990 The ants. Cambridge, MA: Harvard University Press.

[23] Folgarait PJ. 1998 Ant biodiversity and its relationship to ecosystem functioning: a review. Biodiv. Conserv. **7**, 1221-1244. (10.1023/A:1008891901953)

[24] Schultheiss P, Nooten SS, Wang R, Wong MK, Brassard F, Guénard B. 2022 The abundance, biomass, and distribution of ants on Earth. Proc. Natl Acad. Sci. USA **119**, e2201550119. (10.1073/pnas.2201550119) 36122199 PMC9546634

[25] Andersen AN, Majer JD. 2004 Ants show the way Down Under: invertebrates as bioindicators in land management. Front. Ecol. Environ. **2**, 291-298. (10.1890/1540-9295(2004)002[0292:ASTWDU]2.0.CO;2)

[26] Basu P. 1997 Seasonal and spatial patterns in ground foraging ants in a rain forest in the Western Ghats, India. Biotropica **29**, 489-500. (10.1111/j.1744-7429.1997.tb00043.x)

[27] Fellers JH. 1989 Daily and seasonal activity in woodland ants. Oecologia **78**, 69-76. (10.1007/BF00377199) 28311903

[28] Levings SC. 1983 Seasonal, annual, and among-site variation in the ground ant community of a deciduous tropical forest: some causes of patchy species distributions. Ecol. Monogr. **53**, 435-455. (10.2307/1942647)

[29] Queiroz AC et al. 2023 Ant diversity decreases during the dry season: a meta-analysis of the effects of seasonality on ant richness and abundance. Biotropica **55**, 29-39. (10.1111/btp.13158)

[30] Kaspari M, de Beurs K. 2019 On the geography of activity: productivity but not temperature constrains discovery rates by ectotherm consumers. Ecosphere **10**, e02536. (10.1002/ecs2.2536)

[31] Bernstein RA. 1979 Schedules of foraging activity in species of ants. J. Anim. Ecol. **48**, 921-930. (10.2307/4204)

[32] Heller NE, Gordon DM. 2006 Seasonal spatial dynamics and causes of nest movement in colonies of the invasive Argentine ant (*Linepithema humile*). Ecol. Entomol. **31**, 499-510. (10.1111/j.1365-2311.2006.00806.x)

[33] Laskis KO, Tschinkel WR. 2009 The seasonal natural history of the ant, *Dolichoderus mariae*, in northern Florida. J. Insect Sci. **9**, 2. (10.1673/031.009.0201) 19611227 PMC3011848

[34] Lynch JF. 1981 Seasonal, successional, and vertical segregation in a Maryland ant community. Oikos **37**, 183-198. (10.2307/3544464)

[35] Neves FS, Antoniazzi R, Camarota F, Pacelhe FT, Powell S. 2021 Spatiotemporal dynamics of the ant community in a dry forest differ by vertical strata but not by successional stage. Biotropica **53**, 372-383. (10.1111/btp.12918)

[36] Gordon SC, Meadley-Dunphy SA, Prior KM, Frederickson ME. 2019 Asynchrony between ant seed dispersal activity and fruit dehiscence of myrmecochorous plants. Am. J. Bot. **106**, 71-80. (10.1002/ajb2.1214) 30644530

[37] Warren II RJ, Bahn V, Bradford MA. 2011 Temperature cues phenological synchrony in ant-mediated seed dispersal. Glob. Chang. Biol. **17**, 2444-2454. (10.1111/j.1365-2486.2010.02386.x)

[38] Samways MJ. 1990 Species temporal variability: Epigaeic ant assemblages and management for abundance and scarcity. Oecologia **84**, 482-490. (10.1007/BF00328164) 28312964

[39] Bigger M. 1976 Oscillations of tropical insect populations. Nature **259**, 207-209. (10.1038/259207a0)

[40] Berman M, Andersen AN, Ibanez T. 2013 Invasive ants as back-seat drivers of native ant diversity decline in New Caledonia. Biol. Invas. **15**, 2311-2331. (10.1007/s10530-013-0455-6)

[41] Economo EP, Sarnat EM. 2012 Revisiting the ants of Melanesia and the taxon cycle: historical and human-mediated invasions of a tropical archipelago. Am. Nat. **180**, E1-E16. (10.1086/665996) 22673659

[42] Rizali A, Lohman DJ, Buchori D, Prasetyo LB, Triwidodo H, Bos MM, Yamane S, Schulze CH. 2010 Ant communities on small tropical islands: effects of island size and isolation are obscured by habitat disturbance and ‘tramp’ ant species. J. Biogeogr. **37**, 229-236. (10.1111/j.1365-2699.2009.02194.x)

[43] Katayama M, Tsuji K. 2010 Habitat differences and occurrence of native and exotic ants on Okinawa Island. Entomol. Sci. **13**, 425-429. (10.1111/j.1479-8298.2010.00400.x)

[44] Kennedy S et al. 2022 Richness and resilience in the Pacific: DNA metabarcoding enables parallelized evaluation of biogeographic patterns. Mol. Ecol. Early View. (10.1111/mec.16575) 35729790

[45] Yamauchi K, Ogata K. 1995 Social structure and reproductive systems of tramp versus endemic ants (Hymenoptera: Formicidae) of the Ryukyu Islands. Pacific Sci. **49**, 55-68.

[46] Lester PJ, Gruber MAM. 2016 Booms, busts and population collapses in invasive ants. Biol. Invas. **18**, 3091-3101. (10.1007/s10530-016-1214-2)

[47] Kaspari M, Weiser MD, Marshall KE, Siler CD, de Beurs K. 2022 Temperature–habitat interactions constrain seasonal activity in a continental array of pitfall traps. Ecology **104**, e3855. (10.1002/ecy.3855) 36054605

[48] Rowles AD, Silverman J. 2009 Carbohydrate supply limits invasion of natural communities by Argentine ants. Oecologia **161**, 161-171. (10.1007/s00442-009-1368-z) 19452171

[49] Shik JZ, Silverman J. 2013 Towards a nutritional ecology of invasive establishment: aphid mutualists provide better fuel for incipient Argentine ant colonies than insect prey. Biol. Invas. **15**, 829-836. (10.1007/s10530-012-0330-x)

[50] Terayama M, Takamine H, Kubota S. 2009 Ants in Okinawa, pp. 182. Naha City: Hidetsune Takamine.

[51] Terayama M, Kubota S, Eguchi K. 2014 Encyclopedia of Japanese ants, pp. 278. Tokyo: Asakura Shoten.

[52] Ito Y, Takamine H, Yamauchi K. 1998 Abundance and species diversity of ants in Forests of Yanbaru, the northern part of Okinawa Honto with special reference to effects of undergrowth removal. Entomol. Sci. **1**, 347-355.

[53] Suwabe M, Ohnishi H, Kikuchi T, Kawara K, Tsuji K. 2009 Difference in seasonal activity pattern between non-native and native ants in subtropical forest of Okinawa Island, Japan. Ecol. Res. **24**, 637-643. (10.1007/s11284-008-0534-9)

[54] Ross SRP-J, Friedman NR, Dudley KL, Yoshimura M, Yoshida T, Economo EP. 2018 Listening to ecosystems: data-rich acoustic monitoring through landscape-scale sensor networks. Ecol. Res. **33**, 135-147. (10.1007/s11284-017-1509-5)

[55] Van Buskirk J. 2005 Local and landscape influence on amphibian occurrence and abundance. Ecology **86**, 1936-1947. (10.1890/04-1237)

[56] R Core Team. 2023 R: a language and environment for statistical computing. Vienna, Austria: R Foundation for Statistical Computing. (https://www.R-project.org/).

[57] Pebesma E. 2018 Simple features for R: standardized support for spatial vector data. R J. **10**, 439-446. (10.32614/RJ-2018-009)

[58] Hijmans R. 2023 raster: Geographic data analysis and modeling. R package version 3.6-23. See https://CRAN.R-project.org/package=raster.

[59] Kaspari M, Weiser MD, Marshall KE, Miller M, Siler C, de Beurs K. 2022 Activity density at a continental scale: what drives invertebrate biomass moving across the soil surface? Ecology **103**, e03542. (10.1002/ecy.3542) 34614206

[60] Uhler J, Haase P, Hoffmann L, Hothorn T, Schmidl J, Stoll S, Welti EAR, Buse J, Müller J. 2022 A comparison of different Malaise trap types. Insect Conserv. Divers. **15**, 666-672. (10.1111/icad.12604)

[61] Longino JT, Colwell RK. 1997 Biodiversity assessment using structured inventory: capturing the ant fauna of a tropical rain forest. Ecol. Appl. **7**, 1263-1277. (10.1890/1051-0761(1997)007[1263:BAUSIC]2.0.CO;2)

[62] Guénard B, Weiser MD, Gomez K, Narula N, Economo EP. 2017 The Global Ant Biodiversity Informatics (GABI) database: synthesizing data on the geographic distribution of ant species (Hymenoptera: Formicidae). Myrmecol. News **24**, 83-89.

[63] Janicki J, Narula N, Ziegler M, Guénard B, Economo EP. 2016 Visualizing and interacting with large-volume biodiversity data using client–server web-mapping applications: the design and implementation of antmaps.org. Ecol. Inform. **32**, 185-193. (10.1016/j.ecoinf.2016.02.006)

[64] McArdle BH, Gaston KJ. 1995 The temporal variability of densities: back to basics. Oikos **74**, 165-171. (10.2307/3545687)

[65] Gaston KJ, McArdle BH. 1994 The temporal variability of animal abundances: measures, methods and patterns. Phil. Trans. R. Soc. Lond. B **345**, 335-358. (10.1098/rstb.1994.0114)

[66] Hsieh TC, Ma KH, Chao A. 2016 iNEXT: an R package for rarefaction and extrapolation of species diversity (Hill numbers). Meth. Ecol. Evol. **7**, 1451-1456. (10.1111/2041-210X.12613)

[67] Hillebrand H, Kunze C. 2020 Meta-analysis on pulse disturbances reveals differences in functional and compositional recovery across ecosystems. Ecol. Lett. **23**, 575-585. (10.1111/ele.13457) 31943698

[68] Legendre P, De Cáceres M. 2013 Beta diversity as the variance of community data: dissimilarity coefficients and partitioning. Ecol. Lett. **16**, 951-963. (10.1111/ele.12141). 23809147

[69] Dray S et al. 2023 adespatial: multivariate multiscale spatial analysis (0.3-21). See https://CRAN.R-project.org/package=adespatial

[70] Hyndman RJ, Athanasopoulos G. 2021 Forecasting: principles and practice, 3rd edn. Melbourne, Australia: OTexts. (https://OTexts.com/fpp3)

[71] O'Hara-Wild M, Hyndman R, Wang E. 2023 fable: forecasting models for tidy time series. R package version 0.3.3. See https://CRAN.R-project.org/package=fable.

[72] Hallett LM, Jones SK, MacDonald AAM, Jones MB, Flynn DFB, Ripplinger J, Slaughter P, Gries C, Collins SL. 2016 codyn: an R package of community dynamics metrics. Meth. Ecol. Evol. **7**, 1146-1151. (10.1111/2041-210X.12569)

[73] Loreau M, de Mazancourt C. 2008 Species synchrony and its drivers: neutral and nonneutral community dynamics in fluctuating environments. Am. Nat. **172**, E48-E66. (10.1086/589746) 18598188

[74] Hallmann CA et al. 2017 More than 75 percent decline over 27 years in total flying insect biomass in protected areas. PLoS ONE **12**, e0185809. (10.1371/journal.pone.0185809) 29045418 PMC5646769

[75] Seibold S et al. 2019 Arthropod decline in grasslands and forests is associated with landscape-level drivers. Nature **574**, 671-674. (10.1038/s41586-019-1684-3) 31666721

[76] Uhler J et al. 2021 Relationship of insect biomass and richness with land use along a climate gradient. Nat. Comm. **12**, 1-9. (10.1038/s41467-021-26181-3) PMC8511018 34642336

[77] Kishimoto-Yamada K, Itioka T. 2015 How much have we learned about seasonality in tropical insect abundance since Wolda (1988)? Entomol. Sci. **18**, 407-419. (10.1111/ens.12134)

[78] Hillebrand H, Langenheder S, Lebret K, Lindström E, Östman Ö, Striebel M. 2018 Decomposing multiple dimensions of stability in global change experiments. Ecol. Lett. **21**, 21-30. (10.1111/ele.12867) 29106075

[79] Ross SRP-J et al. 2022 Predators mitigate the destabilising effects of heatwaves on multitrophic stream communities. Glob. Chang. Biol. **28**, 403-416. (10.1111/gcb.15956) 34689388

[80] White L, O'Connor NE, Yang Q, Emmerson MC, Donohue I. 2020 Individual species provide multifaceted contributions to the stability of ecosystems. Nat. Ecol. Evol. **4**, 1594-1601. (10.1038/s41559-020-01315-w) 33046872

[81] Krushelnycky PD, Gillespie RG. 2008 Compositional and functional stability of arthropod communities in the face of ant invasions. Ecol. Appl. **18**, 1547-1562. (10.1890/07-1293.1) 18767628

[82] Sanders NJ, Gotelli NJ, Heller NE, Gordon DM. 2003 Community disassembly by an invasive species. Proc. Natl Acad. Sci. USA **100**, 2474-2477. (10.1073/pnas.0437913100) 12604772 PMC151365

[83] Moore JW, Olden JD. 2017 Response diversity, nonnative species, and disassembly rules buffer freshwater ecosystem processes from anthropogenic change. Glob. Chang. Biol. **23**, 1871-1880. (10.1111/gcb.13536) 27761971

[84] Stuble KL, Pelini SL, Diamond SE, Fowler DA, Dunn RR, Sanders NJ. 2013 Foraging by forest ants under experimental climatic warming: a test at two sites. Ecol. Evol. **3**, 482-491. (10.1002/ece3.473) 23531642 PMC3605839

[85] Penick CA, Savage AM, Dunn RR. 2015 Stable isotopes reveal links between human food inputs and urban ant diets. Proc. R. Soc. B **282**, 20142608. (10.1098/rspb.2014.2608) PMC4426608 25833850

[86] Jacquemin J, Roisin Y, Leponce M. 2016 Spatio-temporal variation in ant (Hymenoptera: Formicidae) communities in leaf-litter and soil layers in a premontane tropical forest. Myrmecol. News **22**, 129-139.

[87] Marques TG, Espírito-Santo MM, Neves FS, Schoereder JH. 2017 Ant assemblage structure in a secondary tropical dry forest: the role of ecological succession and seasonality. Sociobiology **64**, 261. (10.13102/sociobiology.v64i3.1276)

[88] Shimoji H, Suwabe M, Kikuchi T, Ohnishi H, Tanaka H, Kawara K, Hidaka Y, Enoki T, Tsuji K. 2022 Resilience of native ant community against invasion of exotic ants after anthropogenic disturbances of forest habitats. Ecol. Evol. **12**, e9073. (10.1002/ece3.9073) 35845378 PMC9272207

[89] La Sorte FA, Tingley MW, Hurlbert AH. 2014 The role of urban and agricultural areas during avian migration: an assessment of within-year temporal turnover. Glob. Ecol. Biogeogr. **23**, 1225-1234. (10.1111/geb.12199)

[90] Leveau LM. 2018 Urbanization, environmental stabilization and temporal persistence of bird species: a view from Latin America. PeerJ **2018**, e6056. (10.7717/peerj.6056) PMC6286803 30564519

[91] Bommarco R, Kleijn D, Potts SG. 2013 Ecological intensification: harnessing ecosystem services for food security. Trends Ecol. Evol. **28**, 230-238. (10.1016/j.tree.2012.10.012) 23153724

[92] Gotelli NJ, Ellison AM, Dunn RR, Sanders NJ. 2011 Counting ants (Hymenoptera: Formicidae): biodiversity sampling and statistical analysis for myrmecologists. Myrmecol. News **15**, 13-19.

[93] Kass JM, Yoshimura M, Ogasawara M, Suwabe M, Hita Garcia F, Fischer G, Dudley KL, Donohue I, Economo EP. 2023 Data and code from: Breakdown in seasonal dynamics of subtropical ant communities with land-cover change. Dryad Digital Repository. (10.5061/dryad.zkh1893fk) PMC10565368 37817591

[94] Kass JM, Yoshimura M, Ogasawara M, Suwabe M, Hita Garcia F, Fischer G, Dudley KL, Donohue I, Economo EP. 2023 Breakdown in seasonal dynamics of subtropical ant communities with land-cover change. Figshare. (10.6084/m9.figshare.c.6856583) PMC10565368 37817591

